# Technologies for Single-Cell Isolation

**DOI:** 10.3390/ijms160816897

**Published:** 2015-07-24

**Authors:** Andre Gross, Jonas Schoendube, Stefan Zimmermann, Maximilian Steeb, Roland Zengerle, Peter Koltay

**Affiliations:** 1 Laboratory for MEMS Applications, IMTEK–Department of Microsystems Engineering, University of Freiburg, Georges-Koehler-Allee 103, Freiburg 79110, Germany; E-Mails: andre.gross@imtek.uni-freiburg.de (A.G.); jonas.schoendube@imtek.uni-freiburg.de (J.S.); stefan.zimmermann@imtek.uni-freiburg.de (S.Z.); maximilian.steeb@outlook.de (M.S.); Roland.Zengerle@imtek.uni-freiburg.de (R.Z.); 2 Cytena GmbH, Georges-Koehler-Allee 103, Freiburg 79110, Germany; 3 Hahn-Schickard, Georges-Koehler-Allee 103, Freiburg 79110, Germany; 4 BIOSS–Centre for Biological Signalling Studies, University of Freiburg, Freiburg 79110, Germany

**Keywords:** single-cell analysis, single-cell handling, single-cell separation, single-cell technologies, flow cytometry, laser microdissection, limiting dilution, microfluidics

## Abstract

The handling of single cells is of great importance in applications such as cell line development or single-cell analysis, e.g., for cancer research or for emerging diagnostic methods. This review provides an overview of technologies that are currently used or in development to isolate single cells for subsequent single-cell analysis. Data from a dedicated online market survey conducted to identify the most relevant technologies, presented here for the first time, shows that FACS (fluorescence activated cell sorting) respectively Flow cytometry (33% usage), laser microdissection (17%), manual cell picking (17%), random seeding/dilution (15%), and microfluidics/lab-on-a-chip devices (12%) are currently the most frequently used technologies. These most prominent technologies are described in detail and key performance factors are discussed. The survey data indicates a further increasing interest in single-cell isolation tools for the coming years. Additionally, a worldwide patent search was performed to screen for emerging technologies that might become relevant in the future. In total 179 patents were found, out of which 25 were evaluated by screening the title and abstract to be relevant to the field.

## 1. Introduction

With regards to heterogeneous cell populations, such as those found in many tumors, the generation and analysis of single cells has an increasing impact on various fields of life sciences and biomedical research [1]. The analysis of heterogeneous cell populations in bulk is only able to provide averaged data about the population, by which important information about a small but potentially relevant subpopulation is possibly lost in the background. Cancer development is based on a complex interrelation of mutations, selection, and clonal expansion resulting in a mosaic out of different subclones within a single tumor [2]. If rare subclones, which lead to only subtle genomic signals, can be detected at all from studying bulk populations, it takes a large sequencing and computational effort [3]. In contrast, the analysis of single cells, representing such a subpopulation, can provide very detailed information—information which may be used for therapeutic decisions in an increasingly personalized medicine. A further need for the analysis of single-cells relates to very rare cells like circulating tumor cells, which are surrounded by billions of normal blood cells and have an increasing clinical impact as a so-called liquid biopsy [4]. However, at present, the isolation and separation of single cells is still a technically challenging task. Main challenges are the yield and quality or in other words the integrity and purity of the cells as well as the throughput and the sensitivity of single cell isolation methods. Today, a large variety of technologies for single-cell separation, isolation, and sorting are already available that are applied according to the scientific objective. These technologies can be briefly classified according to their:

- Level of automation, distinguishing manual methods for cell separation like microscope-assisted picking from automated devices such as fluorescence activated cell sorters (FACS).
- Ability to isolate specific/individual cells, distinguishing statistical methods (*i.e.*, the separation of cells according to a certain statistical probability) from a controlled cell separation (*i.e.*, a cell is specifically selected and confirmed to be single).
- Compatibility with certain application requirements, distinguishing technologies mainly applied for production of monoclonal cell cultures (derived from single cells) from technologies preferably used for single-cell genome/proteome analysis.

In this review, we present the current single-cell isolation technologies in consideration of their compatibility with requirements for downstream life-science applications. In order to identify the most relevant technologies to be presented, a market study of single-cell technologies was conducted by the authors by means of an online survey. After briefly describing the methodology of the market study, the five most-used technologies are reviewed in the following in detail. In addition to these most widely adopted technologies, further emerging technologies were identified through a patent search at the European Patent Office (EPO) for worldwide patents about single-cell separation technologies. Many of these patented technologies are probably not commercialized so far, but nevertheless have the potential to enable advances in the field of single-cell research and might become more relevant and well known in the future. Although this review does not claim to be exhaustive, it might turn out be a helpful guide through the heterogeneous field of state-of-the-art single-cell handling technologies.

## 2. Market Study of Single-Cell Technologies

As part of this work, a market survey about the German market for single-cell technologies has been conducted in the summer of 2014. More than 3000 contacts of potential survey participants have been manually selected as statistical population as described in the following. The criterion for (academic) research contacts to be considered for participation was to be active in the field of cell biology. For company contacts, the criterion was to be listed in the publicly available database for biotechnological companies “Biotechnology Database”, provided by BIOCOM AG, Berlin, Germany [5].

**Figure 1:**
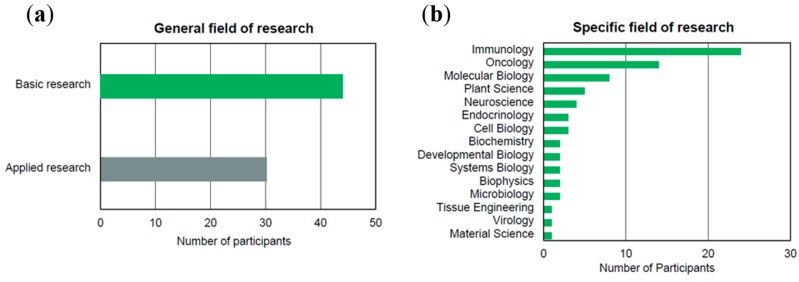
(**a**) Distribution of participants in their general fields of research. 44 participants stated *basic research* and 30 *applied research* as their general field; and (**b**) Distribution of participants in their specific fields of research. *Immunology* and *oncology* were most frequently named.

The data for the marked study was generated by an online questionnaire in June and July 2014. Invitations were sent by e-mail to the previously identified group of contacts. In total 210 participants from German universities, research institutes, and industry have responded to the invitation, out of which 102 have completely filled out the questionnaire. Of all participants 17% are affiliated to universities, 16% to university hospitals, 11% to non-university research organizations, 5% to commercial companies and 51% did not specify their affiliation. Most participants categorized themselves as belonging to the fields of research in medicine (46%) or biology (42%). Figure 1 shows the distribution of the participants in their general and specific fields of research. *Basic research* appears to be slightly more widespread than *applied research*, which can potentially be attributed to the immaturity of the young field of single-cell research. With regards to the more specific research fields, *immunology* and *oncology* were the areas, where most participants worked.

**Figure 2:**
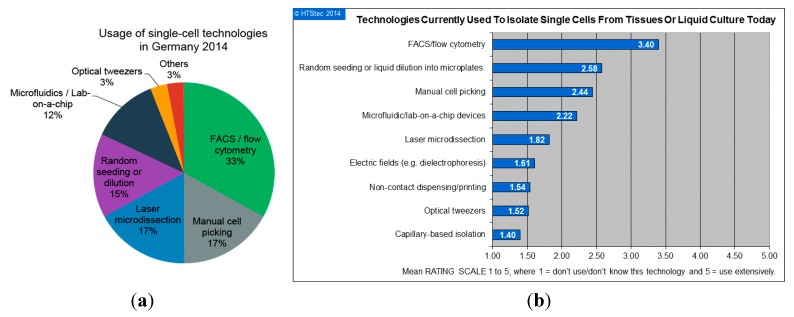
(**a**) The usage of technologies for handling single-cells in Germany in 2014. This data was derived as part of this work by a survey amongst 210 participants from German universities, research institutes and industry; and (**b**) Extensiveness of use of different single-cell technologies (data from “Single Cell Technologies Trends 2014” [6], reproduced with permission from HTStec Limited, Single Cell Technologies Trends 2014, HTStec 2014 URL: http://selectbiosciences.com/ MarketReportsID.aspx?reportID=83).

According to the survey participants, the most commonly used technologies for handling of single cells today are FACS respectively flow cytometry (33%), manual cell picking (17%), laser microdissection (also 17%), random seeding/limiting dilution (15%) and microfluidics/lab-on-a-chip devices (12%). Technologies like optical tweezers and others were mentioned less often (in total 6%). Figure 2a shows the most commonly used technologies in Germany in 2014. The five most prominent technologies will be in the focus of Section 3 of this review. Comparable results were obtained by a worldwide market study performed also in 2014 by HTStec (Cambridge, UK) [6], in collaboration with the authors of this paper. This study ranks the same technologies as the top five most extensively used amongst researchers worldwide (Figure 2b)

A further finding of the survey was, that on average approximately 14 single-cell experiments are performed by the respondents per month, which corresponds to 164 experiments per year. The most frequently given answer was 1–5 experiments per month (mode of the data set). This indicates, that single-cell separation and handling is not a routine procedure yet, but performed regularly by those active in the field.

Finally, the participants were asked to rank the importance of the following criteria for selection of a specific instrument for single-cell isolation: *acquisition costs*, *maintenance & running costs*, *number of cells needed* (minimum to operate the device), *cell viability* (after isolation), *single-cell yield*, *compatibility with existing workflows*, *throughput* (in terms of single cells per second), and *space needed in the laboratory* (for the instrument). It turned out, that all of these criteria are considered to be important (*i.e.*, ranking larger 2.5 out of 5). The lowest ranking had *space needed in the laboratory* (2.75 of 5) and the highest ranking had *cell viability* and *single-cell yield* (4.12 of 5). Certainly, the relative importance of these criteria depend on the specific application, but it is for example noteworthy, that *cell viability* is ranked in average higher than *throughput* (3.52 of 5).

## 3. Single-Cell Isolation Technologies

**Figure 3:**
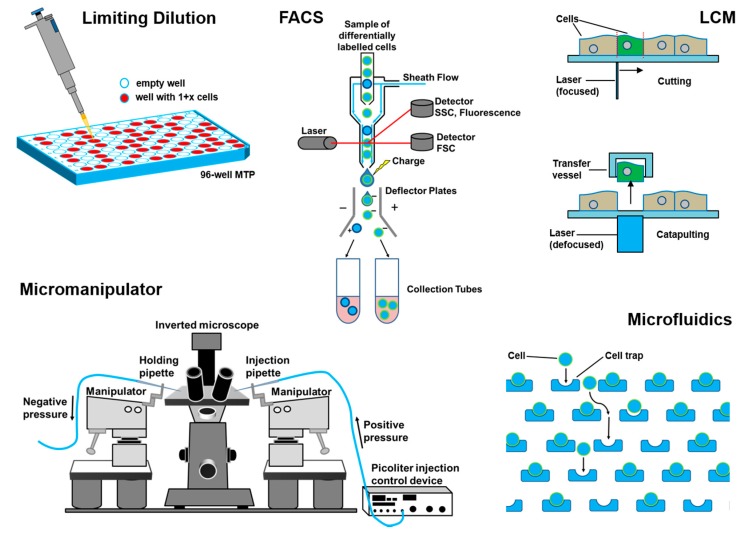
Schematic overview of single-cell separation technologies discussed in the following. The five technologies were identified through market studies as the most commonly used technologies for the handling of single cells (*cf.* (compare to) Figure 1).

Based on the market survey above, the methods and technologies presented hereafter are the most widespread technologies used for single-cell handling. In general, the applied methods strongly depend on the nature and origin of the sample and the processing or analysis to be performed on the cells once being isolated. To illustrate the diversity of sample nature, separation technology, and target applications Figure 3 shows schematics of the working principle of the five methods to be considered in detail in the following.

### 3.1. Flow Cytometry

Thanks to the early pioneers of flow cytometry, since the 1970s researchers have access to ever more powerful flow cytometry instruments. Amongst others, patents and methods developed by Andrew Moldavan 1934 [7], Frank T. Gucker 1947 [8], Wallace H. Coulter 1953 [9], Mark Fulwyler 1965 [10,11], and Wolfgang Dittrich and Wolfgang Göhde 1968 [12] paved the way for the success of commercial flow cytometry [13].

Amongst the various types of flow cytometers, mainly Fluorescence Activated Cell Sorting (FACS) systems provide the ability to isolate single cells, thus they are focus of this section. FACS systems employ laser excitation and offer various analysis options. Cellular properties like relative size and granularity can be extracted as forward scatter (FSC) and side scatter (SSC), respectively. In addition a huge palette of functional properties can be measured by fluorescent staining. In FACS systems, cell suspensions are pressure driven through a flow cell. There they are lined up by a sheath flow liquid exploiting the effect of so called hydrodynamic focusing (see Figure 3). Upon such an arrangement, the cell stream rapidly passes by a laser beam to provide optical excitation and then optical detectors are used downstream to capture cell specific signals. The signals depend on the cells’ respective physical, chemical, or optical properties–often enhanced by synthetic markers such as fluorescent dyes. Apart from size analysis and counting, the bypassing cells can also be sorted. After analysis, the cells are suspended in a closed system of small channels, the cell stream is forced through a small nozzle (typically 60–100 µm orifice diameter) and thereby a liquid jet is formed. By targeted vibrational actuation (e.g., by ultrasound) this jet breaks apart into a continuous stream of free flying droplets some of which carry cells. Using electrically charged plates for deflection of droplets containing cells of interest, these droplets can be guided to a collector vessel (typically a tube or micro well plate).

Popular systems like the FACS-Aria™ III (Becton, Dickinson and Company, Franklin Lakes, NJ, USA) provide up to six different colored excitation lasers and simultaneous fluorescent read-out in up to 18 color channels (FACS-Aria III brochure, BD, 2015, http://static.bdbiosciences.com/documents/ BD_FACSAria_III_brochure.pdf). The system is able to generate up to 100,000 droplets per second and analyze up to 70,000 events per second (FACSAria III technical data sheet, BD, 2015, http://static.bdbiosciences.com/documents/ BD_FACSAria_III_tech_specs.pdf). Similar systems are available from Beckman Coulter (Brea, CA, USA), Sony Biotechnology Inc. (San Jose, CA, USA.), Bio-Rad Laboratories Inc. (Hercules, CA, USA), and others. Typically FACS systems provide different sort modes specialized either on high throughput or enrichment or purity. Depending on the application, type of cells, and the chosen sort mode the actual rate of sorted cells per second can strongly differ between some hundred up to several thousand cells.

Not only since the discovery of hybridoma cells by Koehler and Milstein [14] FACS has become an accepted, worldwide standard in analysis and sorting of cell populations [15], especially due to the known potential hazards in cloning by limiting dilution [16]. Prominent fields of research and application for the FACS technology are for example: DNA content analysis, immunophenotyping, quantification of soluble molecules [17], cell cycle analysis, hematopoietic stem cells, apoptosis, quantification of subpopulations [18], microbial analysis [19], and cancer diagnostics [20,21]. The sample range covers nearly every cell type from blood, bone marrow, tumor, plants, protoplasts, yeast, to bacteria and even viruses.

### 3.2. Laser Capture Microdissection

**Figure 4:**
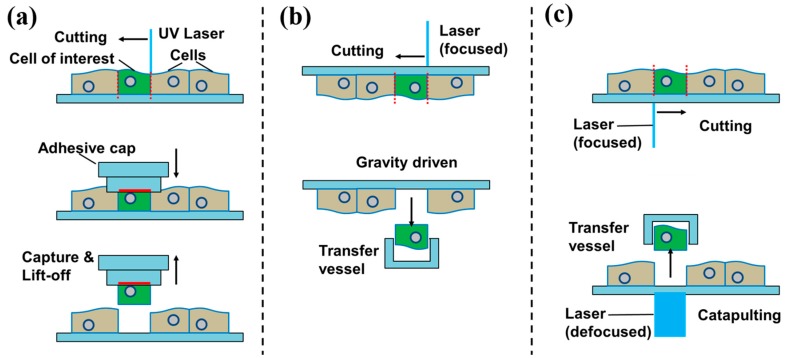
Schematic view on laser capture microdissection (LCM) methods. (**a**) Contact-based via adhesive tapes; (**b**) Cutting with a focused laser followed by capture with a vessel. Cut-out section extracted by gravity; and (**c**) Cutting with a focused laser followed by pressure catapulting with a defocused laser pulse.

Laser capture microdissection (LCM) is an advanced technique to isolate individual cells or cell compartments from mostly solid tissue samples [22,23]. Figure 4 illustrates three different variations of the working principle. A tissue section is observed through a microscope and the target cell or compartment is visually identified. The operator marks the section to be cut off on the display by drawing a line around it. Along this trajectory the laser cuts the tissue and the isolated cell (or compartment) is–if required–extracted. While the cutting procedure using the laser is usually the same, there are several methods to extract the dissected tissue:

Contact-based extraction is done by laser cutting followed by extraction via adhesion, employing adhesive tube caps or heat-absorbing transfer foils, locally made adhesive by infrared (IR) lasers (*cf.* in Figure 4a).

Contact-free gravity-assisted microdissection (GAM) is featuring an inversely mounted substrate placed over a collector tube. Once being cut out by the laser, the target cell (or compartment) falls down into the collector (*cf.* in Figure 4b, Leica LMD7000).

Contact-free laser pressure catapulting (LPC) uses a short defocused laser pulse to ignite a local plasma below the previously cut cell (or compartment) [24,25]. The plasma impulse catapults the cell (or compartment) vertically against gravity into a nearby collector container (*cf.* in Figure 4c, Zeiss PALM MicroBeam LCM).

Samples are typically provided fixed in formalin, embedded in paraffin, or cryo-fixed [26]. Some LCM systems even allow for dissection of living tissue, enabling the extraction of live cells for culture or analysis (Leica LMD7000 with Live Cell Cutting (LCC)).

Analysis of solid tissue is of great interest when investigating heterogeneous tissue sections regarding their cellular structure as well as physiological and pathological processes [27]. In solid tumor research linking the molecular information of individual cells to their specific location in the tissue has become an important research field. Particularly, the access to cells *in situ* is of interest [28]. In combination with immune histological staining, LCM is a powerful tool for solid sample analysis on the single-cell level [29]. In the past years, various applications in single-cell analysis based on LCM extracted cells have been published: Single-cell RT-PCR [30], short tandem repeat analysis (STR) analysis in forensics [31], Western blot and mass spectrophotometry [32]

### 3.3. Limiting Dilution

Today many laboratories and companies use hand-pipettes or pipetting robots to isolate individual cells through dilution of the cell suspension. Due to the statistical distribution of the cells in the suspension, the number of cells in a highly diluted sample can be as low as one single cell per aliquot, when the suspension is split into small volumes (aliquots). This process is termed limiting dilution and is well known for decades for the production of monoclonal cell cultures [33,34,35,36]. Besides antibody production (as done by hybridomas), other applications such as cell-based assays, *etc.* also require cell populations grown from a single-cell.

Such seeding of cells in low concentration is indeed simple to carry out with standard pipetting tools, but it is not very efficient since the probability of achieving a single-cell in an aliquot is of statistical nature. The probability to obtain a certain number of cells per aliquot (*i.e.*, 0, 1, 2, *etc.*) is described by Poisson’s distribution [37]. In order to achieve a sufficiently high probability for the appearance of single cells while at the same time minimizing the probability for multiple cells, the sample has to be strongly diluted. Typically a density of less than one cell per aliquot is applied (e.g., cell concentration smaller than 1 cell/10 µL = 100 cells/mL, if the aliquot volume is 10 µL). Often cited protocols e.g., recommend 0.5 to 0.9 cells per aliquot [16,35,38]. Table 1 presents the respective distribution of cells per well and the ideally achievable number of empty wells, single cells, and multiple cells according to Poisson’s distribution. Obviously, on average only about one third of the prepared wells in a cell culture plate will contain a single-cell. Which of the wells indeed contain single cells has to be confirmed after seeding the cells in a separate process (e.g., by microscopy) due to the statistical nature of the separation method.

**Table 1:** Statistical probability for the number of cells per aliquot according to Poisson’s distribution for cell concentrations of 0.5 and 0.9 cells per aliquot.

0.5 Cells/Aliquot		0.9 Cells/Aliquot	
Cell Number/Well	Probability	Cell Number/Well	Probability
0	61%	0	41%
1	30%	1	37%
2	8%	2	16%
3	1%	3	5%
4	0%	4	1%

### 3.4. Manual Cell Picking

Micromanipulators for manual cell picking typically consist of an inverted microscope combined with micro-pipettes movable through motorized mechanical stages. Micropipettes are made of ultrathin glass capillaries coupled to an aspiration and dispensation unit. The cell sample is typically provided as suspension in a dish or well-plate. Via microscope observation the operator selects a specific cell, moves the micro-pipette in close proximity and aspirates the cell by applying suction to the micropipette. The aspirated liquid volume including the selected cell can be transferred to a collection vessel (e.g., a well of a well-plate), where it is released by dispensation. This process is commonly performed manually.

Micromanipulators enable the controlled separation of selected, living cells from suspension and even allow for isolation of prokaryotic cells [39]. The fields of application range from bacterial analysis [40] to reproductive medicine [41] and forensics [42]

### 3.5. Microfluidics

**Figure 5:**
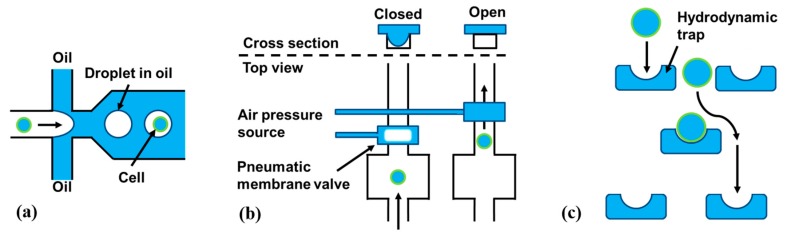
Schematic overview of different microfluidic methods for single-cell isolation. (**a**) An aqueous stream of cells is broken up into individual droplets-in-oil containing random distribution of cells; (**b**) Pneumatic membrane valves use air pressure to close a microfluidic channel by membrane deflection. This stops the flow and can trap a cell; and (**c**) Hydrodynamic traps are passive elements that only fit single cells and hold them at one position.

Vast numbers of microfluidic or lab-on-a-chip devices have been proposed for single-cell analysis and handling in the past years (for reviews see [43] or [44]). In this section, we focus on methods that enable isolation of single cells for further downstream analysis or culture. General cell separation techniques that offer no control at the single-cell level [45] are not within the scope of this section. Though many different microfluidic devices for single-cell separation and handling have been published in the literature, most of these devices use at least one of the three following microfluidic principles to isolate single cells:

- Droplet-in-oil-based isolation as for example published in [46] (Figure 5a);
- Pneumatic membrane valving as for example published in [47] (Figure 5b);
- Hydrodynamic cell traps as for example published in [48] (Figure 5c);

Droplet-based microfluidics uses channels filled with oil to hold separated aqueous droplets (similar to an emulsion). Within these droplets, single cells can be contained and thus be isolated (Figure 5a). Droplet-based microfluidic concepts can separate single cells either randomly according to Poisson’s distribution [46] (similar to the limiting dilution method discussed above) or with even higher yields of over 80% [49]. The biggest advantage of droplets-in-oil-based cell separation and sorting technologies in general is the tremendous throughput of up to several thousand single-cells per second [49]

Pneumatic membrane valves use pressurized air to deflect an elastomer membrane. This membrane deflection closes a microfluidic channel below (Figure 5b). This allows for digitally opening or closing channels in a microfluidic network. Valve-based approaches need a cell detection unit or an operator to isolate cells individually. Typically, these systems are limited in throughput, compared to the droplets-in-oil-technology described before.

Hydrodynamic traps are passive structures in a microfluidic channel that allow only one cell to enter the “trap” (Figure 5). Typically, double occupation is minimized by adjusting the trap size to the average cell size in a given sample. Such systems can operate on a large number of cells in parallel by using a large number of traps [48]. The commercial system C1 from Fluidigm Corp. for example, allows for isolation and subsequent genetic analysis, of up to 96 individual cells in parallel. Hydrodynamic trapping can even be integrated into handheld pipettes to enable manual single-cell pipetting [50] without the need of micromanipulation under a microscope.

**Figure 6:**
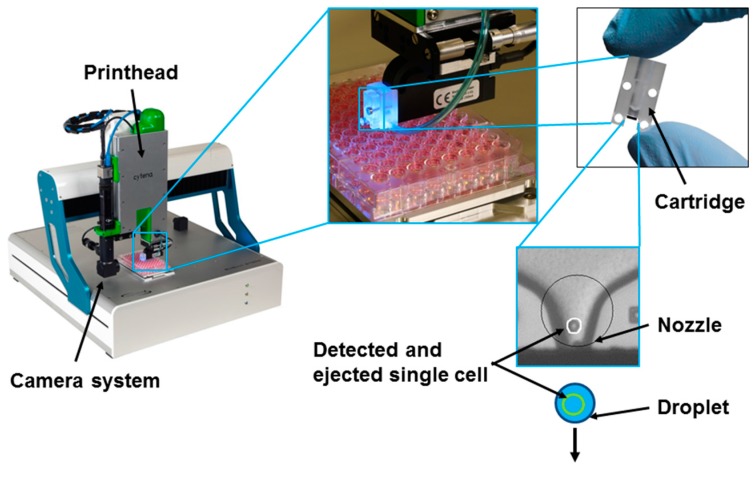
Single-cell printer (SCP) for single-cell isolation. A microfluidic dispenser chip integrated in a polymer cartridge is filled with cell suspension. An automated object recognition algorithm detects cells in the dispenser nozzle prior to the dispensation. This allows for ejection of droplets containing one single-cell only and their deposition in direction of the arrow on various substrates, such as micro-well plates.

Furthermore, approaches to miniaturize flow cytometers by use of microfluidic technologies have been proposed [51]. One of the goals of this field of research is to bring the advantages of flow cytometers (see Section 3.1) such as cell sorting and counting to small and affordable devices, which can potentially be portable. Microfluidic lab-on-a-chip technologies for single-cell applications demonstrate exciting opportunities, but are mostly specifically designed to serve a particular application and therefore exhibit only little flexibility regarding upstream sample preparation and downstream analysis methods. To increase the flexibility and enable easier interfacing with other upstream and downstream methods, the authors of this review have proposed previously a flexible single-cell isolation system (Figure 6), based on inkjet-like single-cell printing [52,53]. This so called single-cell printer (SCP) uses an imaging system and automated object recognition algorithms to detect cells in a microfluidic dispenser chip that can produce droplets similar to an inkjet printer. Cells are classified in the nozzle of the chip and subsequently ejected within a microdroplet (60 µm in diameter) to be deposited onto various substrates. Droplets containing no cell or multiple cells are deflected in flight towards a waste container by vacuum suction. The suitability of the SCP for biomedical applications such as single-cell genomics [54] and clonal cell line production has already been demonstrated [53].

## 4. Patent Search for Single-Cell Separation Technologies

In order to identify emerging single-cell separation technologies currently not well known by the scientific community, a patent search has been performed. The reasoning behind this complementary approach is that a market analysis like described above can only be expected to reveal technologies that are sufficiently well known within the user community, while novel technologies that are probably in a pre-commercialization phase are not necessarily known by this group of persons. Still, such emerging technologies could become relevant or even displace existing technologies in the future. In order to screen for novel technologies, we performed a patent search at the European Patent Office (EPO) in the worldwide database for patents. In detail, we were applying the “Smart search” on the EPO homepage (http://www.epo.org/ searching/free/espacenet.html) using combinations of search termini based on Boolean operations: (txt = “single cell” and (txt = isolation OR txt = separation)) NOT (txt = fuel OR txt = solar). This search led to 179 results, which can be found as a complete list in the supplement (Table S1). The titles and abstracts of the 179 results were carefully reviewed in consideration of their relevance as actual isolation technology for single biological cells. Conversely, the patents considered to be particularly relevant should not represent:

- Only an analysis method of single cells.
- A common method using the term “single cell suspension” without addressing specifically a method for single-cell isolation.
- A cell separation method, which is already established and only part of a patented workflow.
- Other, in this context, irrelevant methods by using the term “cell” in a non-biological context such as for a battery or a chamber in a technical device. Therefore, the terms “fuel” and “solar” were already excluded from the original search from the beginning (see above).

According to these criteria 25 patents were selected from the list of search results based on the information provided in the title and abstract of these patents. The resulting list of relevant patents is presented in Table 2.

**Table 2:** Patents of single-cell isolation technologies identified in the worldwide database of the European Patent Office (EPO).

Title	EPO Publication Number	Reference
Methods for multiplex analytical measurements in single cells of solid tissues	AU2013315409 (A1)	[55]
Single-cell isolation screen adapted with pipettor tip	CN104195036 (A)	[56]
An integrated microfluidic device for single-cell isolation, cell lysis and nucleic acid extraction *	CA2817775 (A1)	[57]
System and method for capturing and analyzing cells *	US2014349867 (A1)	[58]
Single-cell automatic analysis device based on dual-optical-path micro-fluidic chip *	CN203929785 (U)	[59]
Microfluidic devices and methods for cell sorting, cell culture and cells based diagnostics and therapeutics *	US2014248621 (A1)	[60]
High-throughput single-cell imaging, sorting, and isolation *	US8934700 (B2); US2014247971 (A1)	[61]
Automatic single cell analysis method based on microfluidic system *	CN103926190 (A)	[62]
Apparatus for single cell separation and position fixing *	US2013129578 (A1); US8475730 (B2)	[63]
Method and apparatus for single cell isolation and analysis	US2012315639 (A1)	[64]
Apparatus for magnetic separation of cells	US2012045828 (A1)	[65]
Method and apparatus for the discretization and manipulation of sample volumes *	CN102187216 (A)	[66]
Plate for separating single cell	JP2011152108 (A); JP5622189 (B2)	[67]
Array apparatus for separation of single cell *	KR20110037345 (A); KR101252829 (B1)	[68]
Device and method for continuously analyzing single-cell contents by miniflow control chip at high speed *	CN101923053 (A); CN101923053 (B)	[69]
Complete set of equipment for single cell gel electrophoresis test	CN201662556 (U)	[70]
Single cell analysis of membrane molecules *	US2009173631 (A1)	[71]
Single-cell inclusion analytical method based on micro-fluidic chip *	CN101393124 (A)	[72]
Analytical system based on porous material for highly parallel single cell detection *	US2008020453 (A1)	[73]
Single cell isolation apparatus and method of use	US6538810 (B1)	[74]
Cell isolation and screening device and method of using same *	WO03011451 (A1)	[75]
Cell transfer mechanism and cell fusion apparatus *	JPH0731457 (A)	[76]
Device for automatically testing single cell dielectric spectrum based on composite dielectrophoresis	CN201075104 (Y)	[77]
High-pass cell separation device and use method therefor *	CN1962845 (A)	[78]
Cell inclusions analysis method based on microfluid chip *	CN1734265 (A)	[79]

*, indicate patents which can be assigned to the field of microfluidics.

Considering that isolation of biological cells means handling of particles in liquid typically in the range of 10–100 µm in diameter, the majority of these patents (17 out of 25) can be assigned to the field of microfluidics, as indicated by the appearance of “microfluidic” or terms inherent to microfluidics in the title, abstract and/or original document of the respective patents. Since microfluidics has been described in section 3.5 in general terms, the authors refrain from a full discussion of these technologies. The remaining rather “non-microfluidic” technologies utilize a targeted lysis within a tissue [55], a separation sieve for a conventional pipette tip [56], antibody-conjugated magnetic beads [64,65], adjustable permeable wells on a plate [67], electrophoresis [70], tailor-made wells on a membrane [74], and dielectrophoresis [77] for the isolation of single cells.

## 5. Future Potential of Single-Cell Technologies

There are currently several indicators that suggest that the field of single-cell analysis is going to grow further in the coming years. One of them is the recent acknowledgments of the field by the journal *Nature Methods*. The increased interest is supported by *Nature Methods* selecting “single-cell methods” as *method to watch 2011* [80] and “single-cell sequencing” as *method of the year 2013* [81]. The demand for single-cell handling technologies is expected to grow simultaneously with the field of single-cell analysis.

**Figure 7:**
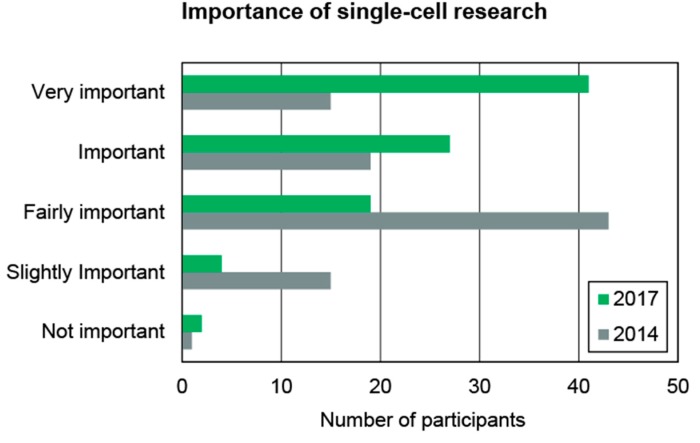
Importance of single-cell analysis to German cell researchers in 2014 and estimated for 2017. This data was derived by a survey amongst 210 participants from German universities, research institutes and industry. A strong growth of interest is expected over the next years.

Figure 7 shows the expected increase in importance of single-cell technologies as seen by the participants of the market study, described in Section 2. In the online survey participants were asked to rank the importance of single-cell research from *not important* to *very important* to their present as well as for their future work. For the present year (2014) the answer with the highest score (mode of the data set) was *fairly important*. For 2017 the expectation with the highest score was *very important* (the highest possible score). These data were acquired amongst German researchers. A similar trend is expected to hold for worldwide researchers, which is in accordance to market data published [6]

Furthermore, participants, who did not have any experience with single-cell technologies in 2014, were asked about their future plans. Out of these, 79% planned to work with single-cell technologies within the next five years. 48% even planned to start working with single-cell technologies in 2015.

In recent years, the field of single-cell analysis has seen a few commercial products hit the market and more are expected to come. Applications currently envisioned or implemented include next generation sequencing (NGS) of single cells [82], isolation of circulating tumor cells for diagnostic purposes [83], or single-cell proteomics [84].

## 6. Discussion

The requirements for technologies to separate and isolate single cells from samples of different nature are as heterogeneous as the purpose for which the cells are used downstream of the separation and isolation process. Depending on the specific requirements, some of the mentioned technologies might be more suitable than others to enable a specific single cell application. In the following, strengths as well as shortcomings of the reviewed commercial technologies are listed and compared to these requirements qualitatively. However, quantitative performance parameters for the various technologies cannot be determined, since this would require focusing on a specific application, which is out of scope of this article. The actual result of a single-cell isolation process—especially with respect to efficiency and cell viability—is depending on many factors like cell type, sample preparation, device calibration, sorting mode, substrate, and many more factors that are hard to quantify in general (e.g., operator skill for manual methods). Thus, the assessment of the different technologies has to remain more general and instead general properties of the technologies like throughput or cell viability, *etc.* are discussed.

### 6.1. General Aspects

Defining general requirements in the field of single-cell handling and analysis is not trivial due to the heterogeneity of the applications mentioned above. The following non-exhaustive list seeks to cover general requirements that many applications have in common.

Sample nature and origin defines to a large extent which technologies can be used at all. The information to be determined from the cells of interest could largely differ: in solid samples often tissue architecture or cell-cell interactions are of interest, while in cell suspensions heterogeneity studies of the cell population are mostly the primary objective.

Cell integrity is often required throughout the cell isolation process. Especially when the genome or proteome is the target of the analysis, cell integrity should be kept prior to lysis to avoid early degradation of DNA/RNA.

Cell viability is required when isolating single-cells for the purpose of production of monoclonal cell cultures or for studying stem cell differentiation. Cells respond to stress factors like mechanical forces, radiation, chemical changes in their environment, *etc.* which can lead to differentiation, reduced viability or even apoptosis. Technologies should provide sufficiently “gentle” extraction and handling when operating on living cells.

Throughput in terms of single cells isolated per second as well as the targeted total number of single cells is another important factor. Especially when large populations with low abundance of target cells are given (e.g., for CTC (Circulating tumor cells) applications), high throughput or high parallelization is mandatory and manual procedures are prohibitive.

Rare samples with a low amount of cells require technologies capable of dealing with small sample volumes and providing low dead volumes. In this context the separation yield (see below) is often a key issue as well in this context to prevent the loss of cells of interest.

Purity of the isolated single cells is crucial when analyzing cellular DNA and RNA. Isolating the cell of interest and while excluding any other contamination from the liquid suspension (e.g., cell fragments, free-DNA, *etc.*) is of highest importance. Transfer volume (the droplet or pipetting volume the cell is typically enclosed in during transfer to the target) and cross-contamination come into play here as well, as they determine the amount of contaminants in the aliquot containing the single cell.

Efficiency concerning yield of the single-cell isolation process can be of importance when using homogeneous samples containing a large number of cells (e.g., for cloning). However, it’s a lot more important when performing single-cell analysis on a rare cell sample or when using complex and costly reagents. Besides the costs per cell that come into play from the economical point of view (e.g., for amplification and library preparation for next-generation sequencing [85]), the analysis of individually selected cells rather than randomly seeded ones or the analysis of a complete population (100% analysis) can impose strict requirements on the separation efficiency. Preventing aliquots that are empty or occupied with multiple cells can be mandatory in certain applications while in others it might be more important that no cell must be lost in the isolation process.

Besides these most relevant general criteria for the assessment of different technologies each application might impose additional specific requirements that have to be carefully considered. For applications with living cells, for example, often carry over-free and sterile operation conditions are requested which calls for technologies relying on disposable components (e.g., microfluidic chips).

Obviously, each single cell separation technology exhibits specific features with respect to the above mentioned aspects that have to be matched to the application under consideration. Usually, this problem is addressed from the side of the application (e.g., single-cell analysis, monoclonal cell cultures, *etc.*) for which the requirements are usually well known. In the following the view point should be shifted towards considering the specific features of the previously discussed technologies.

### 6.2. Flow Cytometry

FACS systems provide high throughput in terms of single-cell analysis and sorting. Paired with high flexibility in terms of cell type, standardized substrates, and sorting modes FACS is a powerful tool. Moreover its suitability for rare cell sorting (subpopulations < 1%) has increasing diagnostic prospect when analyzing heterogeneous cell samples. Some of the FACS systems are able to deposit single cells in micro-well plates with high purity and yield within the time frame of minutes to enable further downstream analysis such as NGS (Next generation sequencing). The popularity and wide-spread use of FACS systems makes them accessible to a broad range of users. 

Nevertheless, for certain applications FACS systems are still limited to some extent. Cells must be in suspension meaning tissues need to be dissociated resulting in loss of cellular functions and cell-cell interactions as well as tissue architecture [86]. Subpopulations with similar marker expression are difficult to differentiate and overlap of emission spectra between fluorochromes may lead to an increasing noise level making low-intensity samples unavailable for detection. Further, FACS sorting may have non-negligible effects on cell viability, which was demonstrated for Chinese Hamster Ovary (CHO) cells and for a human monocytic cell line (THP1) by trypan blue exclusion and necrosis/apoptosis assays [87].

Minimal sample volume for FACS systems is in the range of several hundreds of microliters to milliliters. This is due to the fact, that typically long sections of tubing cause high dead volumes, preventing the use of rare samples, especially when the entire sample needs to be analyzed. And finally, sterile operation is difficult to achieve by FACS systems in general due to the complex system consisting of non-disposable components. However, such limitations are not always present or significant, but strongly dependent on instrument, process parameters (e.g., speed, laser type, *etc.*), cell type, and application.

### 6.3. Laser Capture Microdissection

Whenever single cells need to be isolated from solid samples (e.g., tissue, biopsies) LCM systems are the commonly applied tool of choice. For such applications a single cell’s location within the tissue architecture is essential to know. LCM systems enable separation and isolation of individual cells for downstream analysis. Based on optical microscopes coupled with a coaxial cutting laser and computer assisted control, such systems are relatively easy to handle. The sharply focused pulse lasers cut with sub-micrometer precision and without introducing deleterious heat to the tissue. Another benefit might be that operators actually decide on every cell to select and isolate rather than leaving the decision to automated systems or statistical distributions.

Although modern LCM systems provide a higher level of user-friendliness and automation, the selection and isolation process remains operator based and therefore strongly limits the throughput. The integrity of extracted cells is important for reliable downstream analysis of biomolecules such as DNA, RNA, and proteins [88]. Depending on the quality of fixation [32] and cell extraction method used (adhesive tape, gravity, catapulting) single-cell integrity might be compromised [89]. It might remain unclear, if the cell was actually transferred and/or if any contaminants (e.g., fragments of adjacent cells) were transferred to the substrate along with the cell of interest, especially when using contact-based cell extraction (adhesive cap) [90].

### 6.4. Micromanipulator

Micromanipulators are microscope assisted picking tools allowing for targeted isolation of individual single cells from suspensions, which is a feature not shared by many other technologies. The operator selects the cells to be isolated and performs the aspiration, transfer, and dispensation. Similar to LCM systems, the targeted isolation of a specific cell under microscope vision is one of the key benefits of this technology.

Although being a very flexible technology in terms of cell types and substrates, the serial and manual process of obtaining single cells limits the overall throughput. Furthermore, with the majority of the systems it is not possible to observe and control the correct transfer of the single-cell to its target location. Once the micropipette leaves the microscope’s optical focus plane the transfer volume containing the cell is unobservable as is the actual transition to the target. To actually confirm if a single-cell has successfully been transferred additional observation of the target is required. Recent approaches target improvement on this towards fully automated isolation and placement of single cells assisted by video systems and image processing algorithms [91].

### 6.5. Limiting Dilution

In many pharmaceutical companies, fully automated pipetting robots perform limiting dilution in great numbers at considerable throughput. The process is simple, reproducible and to a certain extent cost-efficient since the degree of automation is very high. However, due to the statistical nature of the process and the lack of control over an individual cell, often further technologies are required downstream to prove the presence of single cells in a specific well, such as automatic microscopic imaging systems. However, limiting dilution remains a simple, gentle, and relatively cost efficient process to obtain single cells with reasonable throughput, but lacking the controlled isolation and sorting as well as proof of single-cell presence. Combined with upstream sorting or enrichment techniques it can constitute an appropriate tool to easily separate viable single cells for downstream analysis.

### 6.6. Microfluidics

Microfluidics plays an increasing role in establishing entire workflows for single-cell separation, isolation, and analysis. Although the number of commercially available systems is still low, this field of research itself is highly dynamic. Microfluidic systems can be operated with very low volumes regarding cell sample as well as reagents, which is advantageous for rare cell applications as well as from an economical point of view. Developed as disposables and once produced with mass-fabrication techniques those systems could provide an attractive alternative, especially with regard to clinical applications: when using disposables, cross-contamination between subsequent samples is not an issue.

Recent hurdles preventing a broader market-entry of microfluidic technologies might be the low degree of flexibility offered by a specific microfluidic chip. Microfluidic systems–unlike the more established technologies discussed before – are often restricted to one single application (e.g., genomic single-cell analysis). Regarding the complexity and variety of single-cell applications throughout the field, and in view of continuously newly established analysis methods, this likely is one of the greatest drawbacks of microfluidic technologies for research applications. Leading scientists and microfluidic companies often regret the absence of a “killer application” supporting their technology (This is true for the entire microfluidic field, not only for those devices targeting single-cell applications.) to enter the markets [92,93,94]. Still, when a sufficiently standardized workflow of isolation, sorting, and analysis can be established by a microfluidic approach, there are probably few other technologies that can provide similar performance in that specific case.

### 6.7. Patents and Emerging Technologies

The patent search performed for this review revealed that 68% (17 out of 25) of the related patents cover microfluidic devices. This patent trend has been present since the middle of the 90s [95] and emphasizes the strong driving forces generated by research and emerging industries. Besides microfluidics few patents deal with alternative technologies e.g., separation by dielectrophoresis or size/affinity based filtering. Partially such alternative technologies are also combined with microfluidic approaches at chip level. Whether or not such special technologies will become relevant in the future depends to a large degree on the applications that can be enabled by them. As outlined before, a strong match between application requirements and technology features can render any technology relevant in the context of a well matching application.

## 7. Conclusions

The review focused on the most frequently used single-cell isolation technologies as derived from a market survey amongst German scientists. It can be concluded that there is no universal technology suitable for all single-cell applications. However, the vast majority of applications can be addressed by at least one of the technologies available today.

Regarding performance FACS systems have the main benefit of high throughput and sorting capability but are cost-intensive and potentially harmful to some cells. LCM is ideal for isolation of single cells from solid tissue and quite unique in this regard. Micromanipulator assisted cell picking is a manual process and therefore slow, but provides maximum control over individual cells. Limiting dilution relies on statistical distribution, is simple to implement and can be automated. However, the presence of single cells often needs to be verified subsequently. Apart from these established technologies, microfluidic technologies allow for integration of entire application specific workflows and only require small amounts of sample and reagents. Thus, microfluidic technologies have potential but are still lacking broad commercial presence, probably due to a lower degree of flexibility. To summarize the topic, Table 3 provides an overview about the key features of the presented technologies.

The worldwide patent search carried out with the objective to identify emerging technologies, resulted in 179 patents out of which 25 have been identified to be particularly relevant to the field. 17 out of those 25 are related to the field of microfluidics, again emphasizing the importance of this evolving field. From the market study it can also be concluded, that the field of single-cell analysis can be expected to grow significantly in the following years and the need for single-cell isolation technologies is likely to increase simultaneously. Presumably, established commercial technologies as well as novel microfluidic devices will contribute equally to advances in this field in the coming years.

**Table 3:** Selected features of discussed technologies. Rating based on the authors’ personal experience and knowledge.

Technology	Automation Level	Throughput	Impact on Cell Integrity	Control over Individual Cell ^1^	Compatibility with Established Workflows ^2^
Fluorescence-Activated Cell Sorting (FACS)	Automatic	High	Often impairing	Yes	High
Limiting dilution	Manual or automatic	Moderate	Gentle	No	High
Micromanipulation	Manual	Low	Moderate	Yes	Moderate
Laser-capture microdissection	Manual	Low	Often impairing	Yes	Low
Microfluidics (Lab-on-a-Chip)	Automatic	Low to high	Diverse	Typically not	Low
Microfluidics (inkjet-like printing)	Automatic	Moderate	Gentle	Yes	High

^1^, Possibility for active selection of single cells before their isolation in contrast to random distribution of individual cells; ^2^, Compatibility with commercially available substrates such as microtiter plates, tubes, slides, *etc.*

## Supplementary Materials

Supplementary materials can be found at http://www.mdpi.com/1422-0067/16/08/16897/s1.
